# Risk assessment of neuromuscular stimulation by energy-based transurethral resection devices: an ex vivo test standard

**DOI:** 10.1186/s12894-020-00630-5

**Published:** 2020-05-27

**Authors:** Ulrich Biber, Ovidiu Jurjut, Markus D. Enderle, Wilhelm K. Aicher

**Affiliations:** 1 Erbe Elektromedizin GmbH, Waldhoernlestrasse 17, 72072 Tuebingen, Germany; 2 Department of Urology, University Hospital Tuebingen, Tuebingen, Germany

**Keywords:** Electrical stimulation, Electrosurgical unit, Radio-frequency current, Compound action potential, Bipolar loop electrode, Bladder, Prostate, Obturator nerve, TURB, TURP

## Abstract

**Background:**

During transurethral resection of bladder tumours (TURB), radio-frequency (RF) currents can lead to adverse neuromuscular stimulation (NMS). Here we present a novel ex vivo method to determine the risk of RF generators and their bipolar TURB modes to cause NMS. We aimed to develop an experimental platform for safety evaluation of new RF generators and their modes with a newly established test standard, suitable for replacement or reduction of animal testing.

**Methods:**

We tested four contemporary RF generators with their bipolar modes for TURB in saline. A two-stage ex vivo approach was pursued: First, we recorded voltages at possible positions of the obturator nerve behind a porcine bladder wall in a TURB model using 18 RF applications per generator. Second, these voltage records were used as stimuli to evoke nerve compound action potentials (CAPs) in isolated porcine axillary nerves. The NMS potential was defined as the ratio between the observed area under the CAPs and the theoretical CAP area at maximum response and a firing rate of 250 Hz, which would reliably induce tetanic muscle responses in most human subjects. The measurement protocol was tailored to optimise reproducibility of the obtained NMS potentials and longevity of the nerve specimens.

**Results:**

As prerequisite for the clinical translation of our results, the robustness of our test method and reproducibility of the NMS potential are demonstrated with an excellent correlation (*r* = 0.93) between two sets of identical stimuli (*n* = 72 each) obtained from 16 nerve segments with similar diameters (4.2 ± 0.37 mm) in the nerve model. The RF generators differed significantly (p < 0.0001) regarding NMS potential (medians: 0–3%).

**Conclusions:**

Our test method is suitable for quantifying the NMS potential of different electrosurgical systems ex vivo with high selectivity at a reasonable degree of standardization and with justifiable effort. Our results suggest that the clinical incidence of NMS is considerably influenced by the type of RF generator. Future generations of RF generators take advantage from the proposed test standard through higher safety and less animal testing. Health professionals and treated patients will benefit most from improved RF surgery using generators with a low NMS risk.

## Background

The surgical application of RF current can lead to adverse NMS. This is commonly attributed to the so called faradic effect created by unwanted frequencies below 20 kHz (pp. 518–519 in [1]). In urology, adverse NMS events have been reported during transurethral resection of bladder (TURB) [2–5] and prostatic tumours (TURP) [4, 6]. Moreover, in gynaecology, the hysteroscopic transcervical resection should be mentioned. TURB is the most common surgical technique (gold standard) for the definitive diagnosis and initial treatment of bladder cancer [5], which is one of the most common urinary tract malignancies [7]. Potential intraoperative complications of TURB include bleeding and bladder perforation, typically as a consequence of stimulation of the obturator nerve, which may be associated with sudden, strong contractions of leg adductors. These contractions can lead to uncontrolled movements of the instruments introduced into the bladder, possibly leading to a bladder perforation, which requires conversion to open surgery. Current in vivo or clinical techniques for evaluating NMS typically include documenting the incidence of adverse NMS events such as obturator nerve reflex/adductor muscle contraction or bladder injury/perforation rate [2, 3, 5, 8, 9]. In some studies, the severity of intraoperative NMS events is evaluated on a rating scale, such as “no”, “weak/moderate” and “strong/severe” [10, 11]. Moreover, compound muscle action potentials from adductor muscles were examined in patients undergoing TUR surgery [12, 13]. To reduce the risk of NMS, several measures were described: First, a pharmacological block of the obturator nerve [8, 11–14]. Second, intubation anaesthesia and relaxation of skeletal muscles of the patient (chpts. 11.3 & 11.3.4 in [15]). Third, a low-power setting, e.g. 50 W for cutting and 40 W for coagulation instead of a typically higher setting [2]. Fourth, the use of bipolar instead of monopolar RF current: Some studies reported advantages of bipolar over monopolar TURB, including a lower recurrence rate, better quality of biopsy specimens for the pathologist, less haemorrhage, lower incidence of the TUR syndrome and also reduced risk of NMS [2, 10]. A recent meta-analysis including seven clinical trials suggests that the incidence of obturator reflex and bladder wall perforation for monopolar vs. bipolar TURB is 14.8% vs. 3.7% (*p* < 0.0001) and 4.3% vs. 0.3% (*p* = 0.003) [3]. However, there are also studies showing that bipolar is not superior to monopolar TURB with respect to these complications [5, 9, 16, 17]. Consequently, the EAU guideline (Update 2016) to “Non-muscle-invasive Bladder Cancer” states that the results concerning resection techniques for TURB remain controversial (chpt. 5.11.3 in [18]). Overall, the measures described to reduce the incidence of NMS do not really solve the problem, as they either pose a different risk to the patient or impair performance of the RF application. A satisfactory solution would be RF generators which, due to technical advances, trigger less NMS. Therefore, we developed an ex vivo test system to assess the unfavourable NMS risk of different RF generators. This was previously only possible with in vivo animal testing or clinical trials.

## Methods

**Fig. 1:**
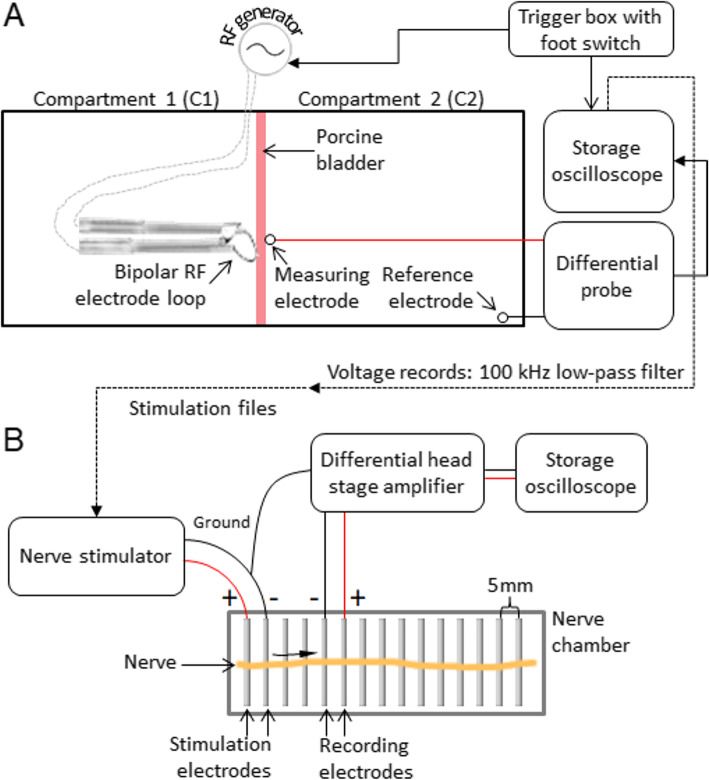
Experimental setup. **a** Ex vivo TURB model – C1 represents the inside and C2 the exterior of the bladder. The tank for the two compartments was made of transparent material and included an exchangeable two-piece tissue holder frame (aperture: 13.5 cm) creating a waterproof seal between C1 and C2 using sealing hoses. Voltage recording was performed at 5 MS/s with a storage oscilloscope. Activation of the instrument and data logging were synchronised with a trigger box which in turn was activated by a foot switch. **b** Ex vivo nerve model – CAP recording at a distance of 1.5 cm from the stimulation site using a differential head stage amplifier and a storage oscilloscope. The nerve chamber’s electrodes were installed at a fixed distance of 5 mm

A two-stage ex vivo approach was pursued: First, during the application of RF energy in an ex vivo TURB model, we recorded voltage curves at possible positions of the obturator nerve behind a porcine bladder wall (Fig. 1a). Second, these voltage records were used as stimuli in another ex vivo model with peripheral porcine nerves (Fig. 1b). The influence of these stimuli on nerve activity, i. e. the generation of evoked nerve compound action potentials (CAP) was recorded. A CAP is the spatial sum of action potentials generated by all stimulated axons of a whole nerve or a large population of axons recorded extracellularly. Thus, in this study, the firing rate and strength of CAPs represent a surrogate measure for the risk of NMS. We refer to this measure as “NMS potential”, which is defined mathematically in subsection “Data analysis”. The tested RF generators (250–450 kHz base frequency) were used with the control algorithm/mode for cutting with bipolar instruments in saline solution. Activation is divided into an ignition phase, in which spark formation is triggered at relatively high current flow (e.g. 8 A), and a cut phase, in which sparking is maintained at relatively low currents (e.g. 4 A). The ignition phase is fixed, whereas during the cut phase the intensity can be adjusted via the generator setting. Since the hardware components and modes differ, the exact electric parameters and instrument-specific adaptations are quite different between manufacturers. Thus, the recorded voltage curves represented a spectrum of different manufacturers and generator settings.

### Experimental design

We tested four different contemporary RF generators (Erbe Elektromedizin GmbH, Bowa-electronic GmbH, Olympus Corporation) combined with a resection electrode loop (24 Fr, WA22507D, Olympus), hereafter called instrument, at their minimum, default and maximum effect settings. Each combination of RF generator and effect setting was applied six times in the ex vivo TURB model. The resultant 72 stimuli were applied twice in the ex vivo nerve model, i. e. 144 values representing an NMS potential were obtained.

### Experimental setup – ex vivo TURB model

This first stage of the ex vivo model was designed to mimic the essential components of TURB surgery (Fig. 1a). We recorded voltage curves at possible positions of the obturator nerve. Porcine bladder tissue separated two relevant functional compartments: One compartment for the instrument (C1), representing the inside of the bladder (mucous layer), which was filled with normal saline solution as during bipolar TURB. The other compartment (C2), representing the exterior of the bladder (adventitia/serous layer), was filled with normal saline as well. In C1 the bladder tissue was cut vertically. The measuring electrode was positioned in C2 contacting the exterior bladder tissue. The distance between instrument and measuring electrode was set to ~ 3 mm in the same horizontal plane given a typical mean bladder tissue thickness of 2.5 mm. However, this distance varied due to the cutting movements (~ 1 cm) of the instrument (in theory ~ 3–6 mm). The distance of ~ 3 mm (or more) was chosen for two reasons: First, it represents a relative safety distance regarding the risk to cause thermal damage to nervous structures [19]. Second, this distance represents a reasonable compromise between sufficient and too much voltage amplitude measured with our equipment. The instrument was fixed to a height gauge for positioning and for cutting the bladder tissue by manual traction. For more accurate positioning of the instrument in C1 a LED light source with an optical fibre was fixed below the measuring electrode in C2, pointing towards the bladder tissue. The spot light shined through the bladder wall allowing for accurate initial instrument positioning in C1. The differential probe for voltage recording (TA058, Pico Technology) was connected to the measuring electrode (point electrode, diameter: 1 mm, surface material: gold) and to a reference electrode, which was fixed at the edge of C2 (Fig. 1a).

### Conduction of test – ex vivo TURB model

Fresh abattoir porcine bladder was stretched evenly in a tissue holder frame. The thickness of the stretched bladder in the retractor frame was measured with a calliper. If necessary, this procedure was repeated to achieve a final thickness of 2–3 mm across the entire aperture. For each RF application, instrument and measuring electrode were repositioned to start over with unused tissue. The instrument was placed in an upright position (electrode tip facing downwards) tilted by 20 ± 5° with respect to the plane of the bladder and was activated with contact to the bladder. The activation time was 900 ± 20 ms with a vertical cut length of ~ 1 cm resulting in an average cut speed of 1.1 cm/s. The cut depth was adjusted to avoid perforation of the bladder while at the same time ensuring a not too superficial cut with a clearly visible trace. In case of perforation the bladder tissue was replaced and the respective measurement was excluded. In case of poor cut quality (no or too superficial cut) the respective measurement was also excluded. RF generator type and effect setting were documented and the voltage record was saved. A “stimulus ID” was assigned to measurements selected for nerve stimulation.

### Pre-processing of voltage records for ex vivo nerve model

Voltage records obtained in the ex vivo TURB model were processed with Scilab software v.6.0.1 (94,528 Rungis, France) for nerve stimulation. Frequencies above 100 kHz that cannot evoke CAPs [20, 21] were removed with a second order low-pass Butterworth filter (Scilab functions: ‘analpf’, ‘dscr’, ‘flts’). This includes the RF generator’s base frequency components, which would have introduced a detrimental noise source and also could have caused thermal damage to the nerves. The processed voltage records, hereafter called stimuli, were sorted in ascending order with respect to peak to peak voltage and divided into groups of six. Additionally, a snippet of a specific stimulus was chosen as reference stimulus, which clearly evoked a CAP and marked the 80% quantile of peak to peak voltages of all stimuli.

### Experimental setup – ex vivo nerve model

The second stage of the ex vivo model used standard electrophysiological methods (Fig. 1b). Isolated peripheral porcine axillary nerve trunks were harvested immediately post mortem and stored in Ringer’s solution (Fresenius Kabi). They were used for CAP recording after an initial storage period of at least two hours but no longer than 32 h. The isolated nerve specimens were placed on electrodes in a nerve chamber that could be closed with a lid to preserve humidity inside. The electrodes in the nerve chamber were used either as stimulation or recording locations. The stimulation electrodes were connected to an arbitrary waveform generator (Keysight 33512B), serving as nerve stimulator. The recording electrodes were connected to a differential head stage amplifier (npi electronic EXT-02F/2). In turn, this amplifier was connected to a storage oscilloscope for CAP recording.

### Conduction of test – ex vivo nerve model

The measurements were performed at ambient temperature. Fixed settings for the differential head stage amplifier were used: 100 Hz high-pass, 20 kHz low-pass and 500× gain. The storage oscilloscope was also used with fixed settings: 1 s sampling duration, 1 MS/s sampling rate. The measurement protocol for the CAP recording consisted of the following steps:

1. Determination of maximal CAP amplitude:
  - Stimulation using a conventional rectangular pulse with a duration of 100 μs at a stimulus amplitude of 7 V (about 2× higher than the typical stimulus intensity required to evoke the maximal CAP amplitude)
  - If the maximal CAP amplitude was < 4 mV, the recording location was changed
2. Determination of stimulus amplitude (threshold) to evoke a just measurable CAP (~ 40 μV)
  - Stimulation by conventional rectangular pulse with a duration of 100 μs
  - Adjustment of stimulus amplitude starting with 100 mV
  - If the threshold was > 200 mV, the recording location was changed
3. Calculation of 80% of the maximal CAP amplitude ➔ CAP_80_
4. Adjustment of reference stimulus to evoke CAP_80_ to obtain a scaling factor (SF):
  - For example: $$ SF=\frac{\ \mathrm{Reference}\ \mathrm{stimulus}\ \mathrm{amplitude}\ \mathrm{to}\ \mathrm{evoke}\ {\mathrm{CAP}}_{80}:7.1\ \mathrm{V}}{\mathrm{Original}\ \mathrm{reference}\ \mathrm{stimulus}\ \mathrm{amplitude}:5.7\ \mathrm{V}}\approx 1.25 $$
5. Stimulation with one set of six stimuli scaled by the SF calculated in Step 4
6. Stimulation with reference stimulus scaled by the SF of Step 4 to check if
  - CAP amplitude > 80% × CAP_80_ ➔continuation with Step 5
  - CAP amplitude < 80% × CAP_80_ ➔continuation with Step 7
7. Placement of nerve specimen in Ringer’s solution for at least 1 min
8. Placement of nerve specimen in nerve chamber at former position as exactly as possible
9. Continuation with Step 1 until all stimuli were recorded

The measurement protocol was conducted twice with all 72 stimuli. Additionally, for control and background measurements, the same stimuli were applied on non-functional nerve specimens with the respective scaling factors calculated in Step 4 of the measurement protocol. To render a nerve specimen non-functional it was squeezed with tweezers between stimulation and recording locations. After each measurement “stimulus ID” and amplitude were documented and the CAP recording was saved.

### Data analysis

Data was analysed with Scilab. The NMS potential was calculated for each stimulation in the nerve model: First, DC offset was removed and the records were high-pass filtered at 100 Hz (linear phase FIR-filter, Scilab functions ‘wfir’, ‘syslin’ and ‘flts’) and low-pass filtered at 3 kHz (Second order Butterworth, Scilab functions ‘analpf’, ‘dscr’ and ‘flts’). Second, background measurements were subtracted from the respective test measurements to eliminate signal distortions due to stimulus artefacts. Third, these optimized CAP records were used for the automated CAP detection (Figure S1). The areas under the CAPs were used to calculate the NMS potential of each individual stimulation:

$$ NMS\kern0.3em pote\mathrm{n} tial=\sum \limits_{n=1}^{No. of\kern0.34em CAPs}\frac{1}{N\_{\operatorname{MAX}}_{CAPs}}\times \frac{Area\kern0.3em of\kern0.3em CAP(n)}{Area\kern0.3em of\kern0.3em \mathit{\max}.\kern0.3em amplitude\kern0.3em CAP} $$

The NMS potential was defined relative to the CAP, which is a well-established measure for nerve activity [22–25]. An NMS potential of 100% corresponds to the maximum CAP area at a firing rate of 250 Hz, which would reliably induce tetanic muscle responses in most human subjects ([26], see their Fig. 5), although 50 Hz are typically sufficient for a single muscle fibre [27]. We have therefore assumed 250 Hz as worst-case firing rate of the CAPs, i. e. the worst-case number of CAPs during the analysed time window of 850 ms (N_Max_CAPs_) was set to 213. The actual number of evoked CAPs for a stimulus (*No. of CAPs*) and their areas were set in relation to N_MAX_CAPs_. This was achieved by adding up the areas of the evoked CAPs (*Area of CAP(n)*) and dividing it by the area that corresponds to 213 (N_MAX_CAPs_) CAPs with maximal amplitude. Finally, the NMS potential was expressed as percentage to emphasize the relation to the worst-case stimulation. Thus, the NMS potential ranged from 0 to 100% and has no unit. In theory, NMS potentials higher than 100% could occur but it surely includes the worst-case stimulation for the stimuli applied. The highest NMS potential actually obtained was 17%.

Statistical calculations were performed with GraphPad Prism software v.7.01 (GraphPad Software, California, U.S.A.). Median, range and confidence limits were calculated for descriptive purposes. Differences between continuous data of more than two samples were detected using one factor analysis of variance with post hoc multiple comparisons. The normal distribution was confirmed or disconfirmed with the Kolmogorov-Smirnov-Test for small sample size. Differences between NMS potentials were detected using the Kruskal-Wallis test since it was not normally distributed. For analysing differences between specific groups corrected Dunn’s test was used. Linear regression analysis was used to describe correlations (NMS potentials from identical stimuli).

## Results

### CAPs in different phases of RF activation

**Fig. 2:**
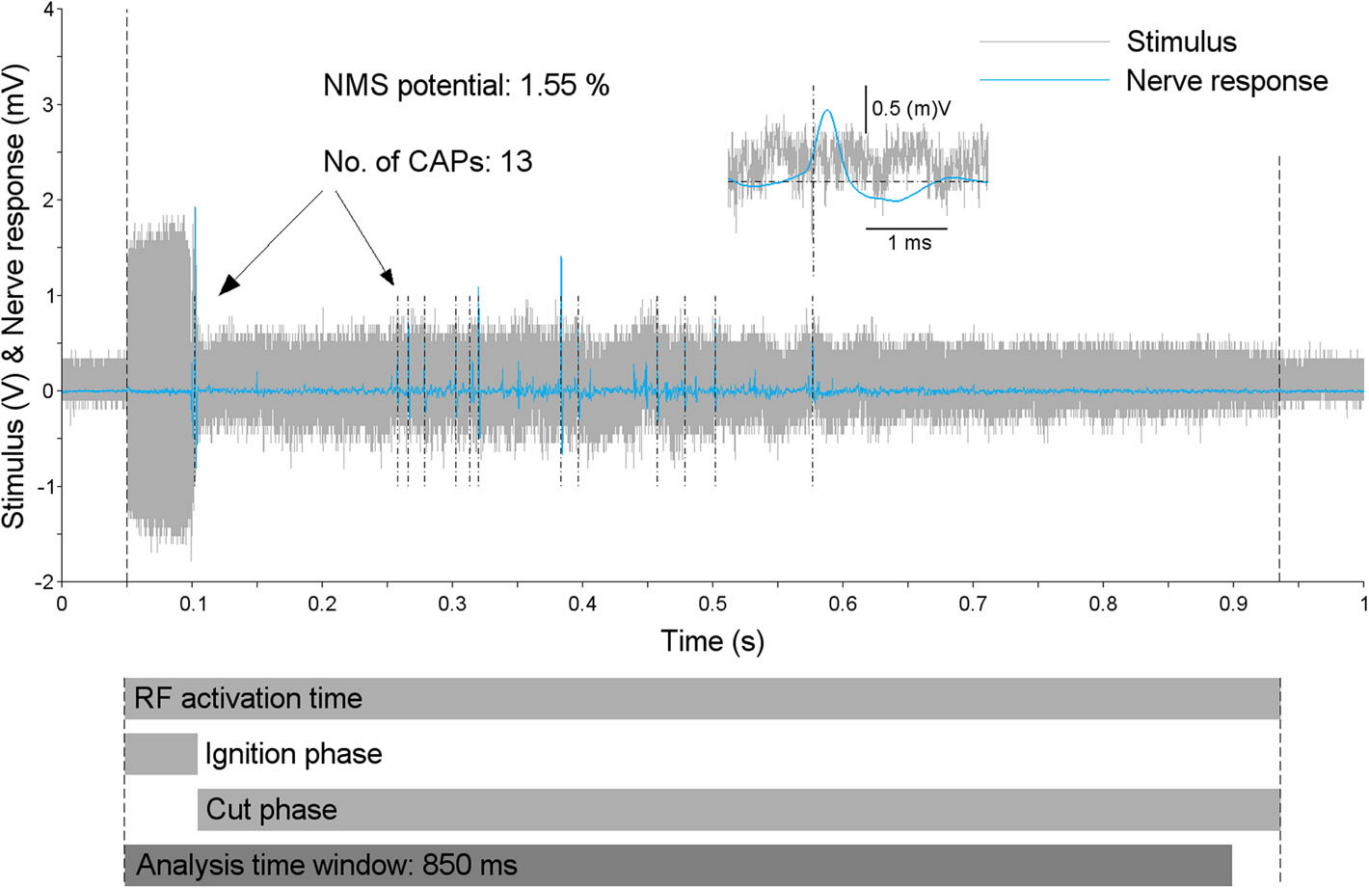
Typical example of nerve response (blue) to a single stimulus (grey) that was applied to an isolated porcine axillary nerve and originates from an RF activation with a bipolar resection electrode loop. Amount and strength of detected CAPs within the analysed time window defined the NMS potential. See Figure S1 for details on the CAP detection algorithm. CAPs were detected both during the ignition and the cut phase of the RF activation corresponding to the stimulus. The zoom view shows the last CAP detected in this stimulation

In a simplified view the activation of bipolar RF instruments in saline is characterised by two distinct phases: In the first phase the ignition of a spark is triggered by relatively high current flow and during the second phase, where cutting takes place, the spark is sustained at a relatively lower current flow. The ignition and cut phases can be distinguished in the stimulus traces corresponding to the RF activations, e.g. in Fig. 2. Both during the ignition and the cut phase CAPs were detected. For this, the stimulus shown in Fig. 2 represents a typical example where one or a few relatively strong CAPs occur when the spark is ignited and several, usually weaker CAPs occur when the electrode loop is brought closer to the possible nerve position. In the definition of the NMS potential, this distinction is not made but it is important to take into consideration that these two contributions are included.

### Inter data-set reliability

**Fig. 3:**
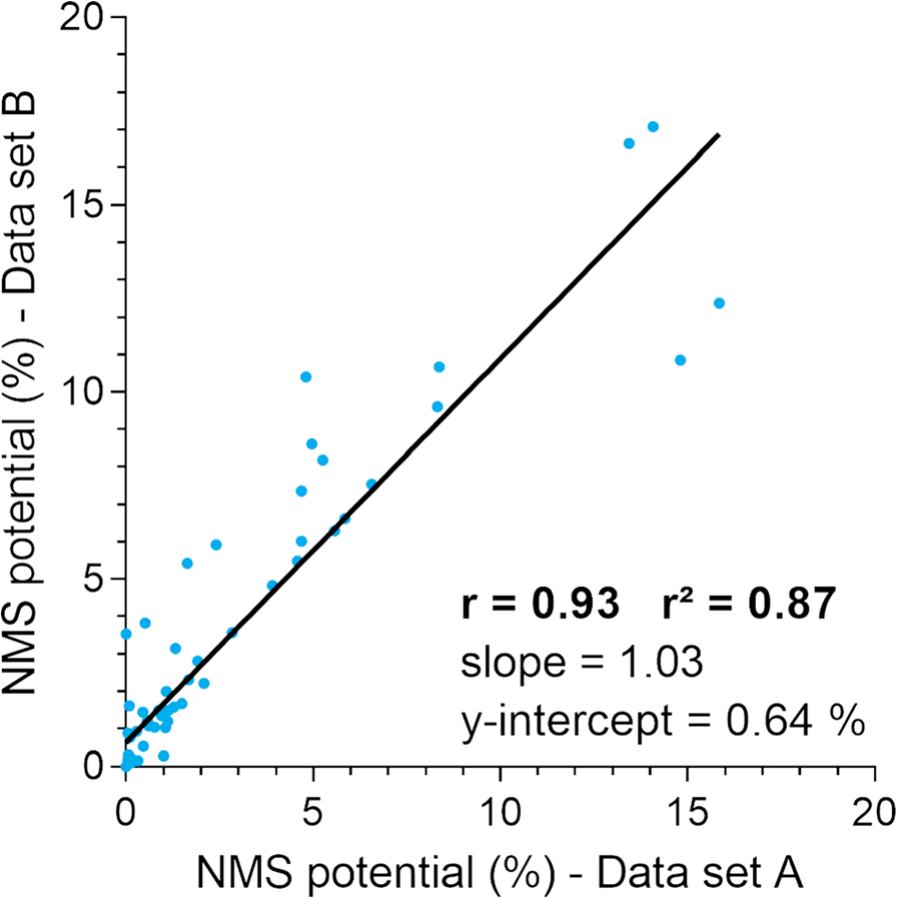
Correlation of NMS potentials obtained from 16 porcine axillary nerve segments in response to two identical sets of stimuli (*n* = 72 per data set) originating from RF activations with a bipolar resection electrode loop. ~ 76% (55/72) of the stimuli resulted in a NMS potential larger than 0%

An important feature of our two-stage approach is that stimuli obtained from the first stage might be applied multiple times in the second stage. Applying exactly the same stimuli multiple times allows demonstrating reproducibility of the NMS potential. Furthermore, with respect to standardisation and reproducibility two variables that define the physiological status of the nerve specimens are most important: First, the number of undamaged fibres and, second, the responsiveness of these fibres. Therefore, it was important to harvest the nerve specimens carefully without imposing mechanical loads and to use similar nerve diameters (target diameter: 4 mm). The 16 nerve segments used actually had an average diameter of 4.2 ± 0.37 mm. Consequently, a similar amount of undamaged nerve fibres per specimen can be assumed. Moreover, the responsiveness of the specimens was monitored according to Step 1 and Step 2 of the measurement protocol. A correlation plot of the two data sets (Fig. 3), in which exactly the same stimuli (*n* = 72) were applied twice, shows a high degree of correlation (Spearman *r* = 0.93) and a very robust goodness of fit to a linear regression (*r*^2^ = 0.87). The slope of the regression line (1.03) was not significantly different from one (*p* = 0.60) and the y-intercept was close to zero (0.64%).

### NMS potential of different RF generators

**Fig. 4:**
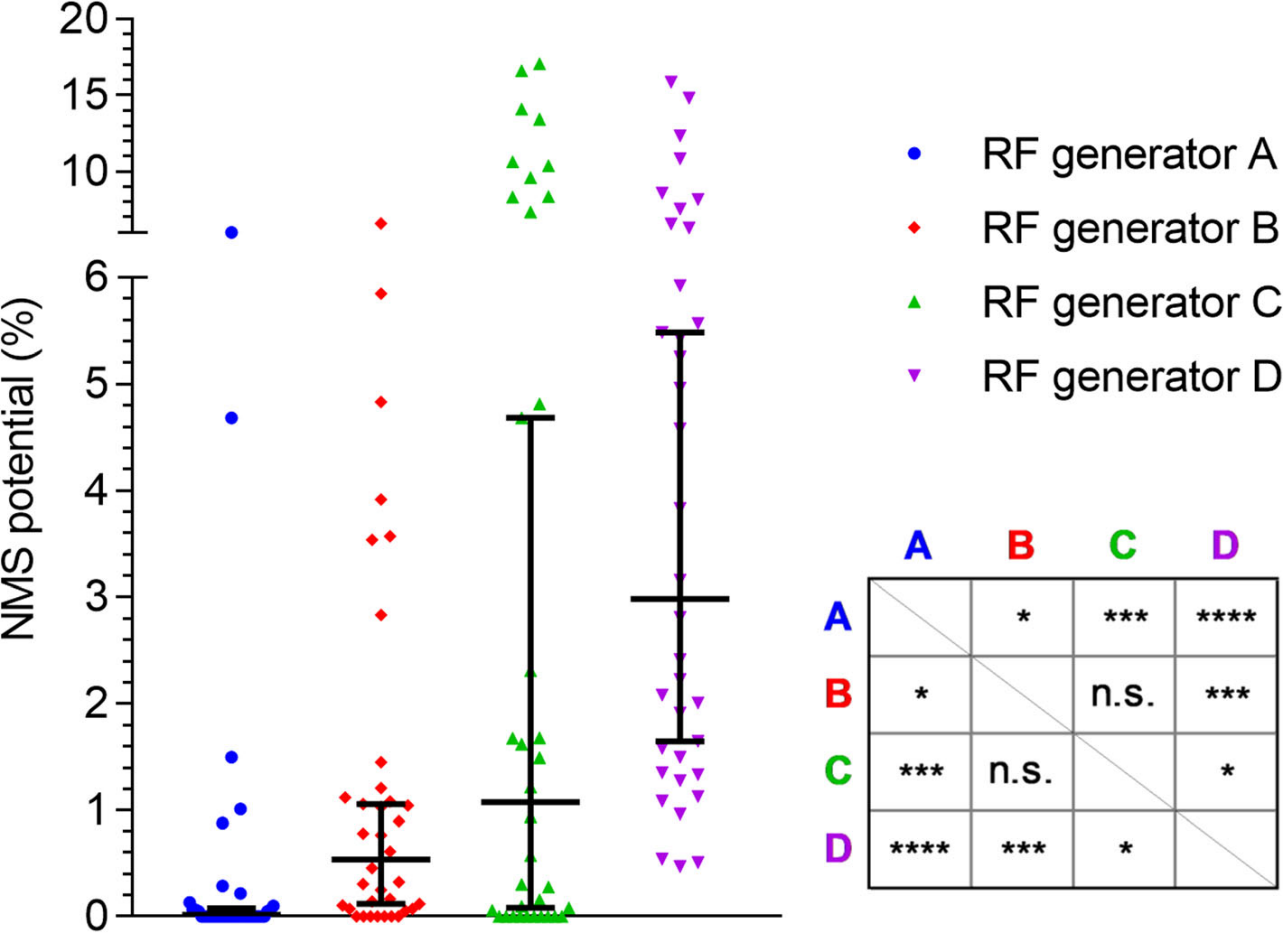
NMS potentials of four contemporary RF generators (*n* = 36 per generator) with medians and 95% CI (error bars) and post-hoc table (Dunn’s test). The NMS potentials of each generator originate from RF activations with a bipolar resection electrode loop used in saline with minimum, default and maximum effect settings in equal shares (*n* = 12 per effect setting)

In the ex vivo nerve model we tested 72 stimuli from four contemporary RF generators, i. e. 18 stimuli from each generator. Each stimulus was tested twice on separate nerves. Therefore, 144 NMS potential readings were obtained. We found significant differences in NMS potentials (Fig. 4) between the four commercial RF generators (Kruskal-Wallis test *p* < 0.0001) with medians between 0% (RF generator A) and ~ 3% (RF generator D) (Table 1).

**Table 1:**
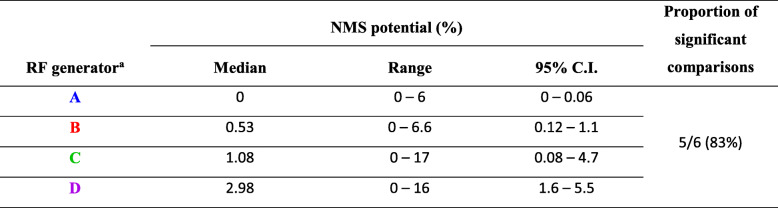
NMS potential of four contemporary RF generators

^**a**^with bipolar resection electrode loop in saline solution with minimum, default and maximum effect settings

## Discussion

The basis for the validity of the ex vivo TURB model was to mimic the essential components of this clinical application. The usage of normal saline in compartment C1 is equivalent to the clinical setting of TURB. The usage of normal saline in compartment C2 is a simplification based on multiple considerations: First, the electrical conductivity of normal saline solution is quite similar to that of extracellular human blood plasma or interstitial fluid outside the bladder wall (~ 1.9 S/m and 1.7 S/m at 25 °C). Second, exactly matching the conductivities is not mandatory because the extracellular fluid conductivity may vary, e. g. as a consequence of various pathological states [28]. Third, assuming the relatively high conductivity of normal saline solution within C2 is an appropriate worst-case estimate, in which a fluid behind the bladder wall makes direct contact to the obturator nerve without further tissue in between. In comparison, muscle or connective tissue is approximately five times less conductive (0.3–0.4 S/m). The usage of porcine bladder is the most appropriate substitute for human bladder since pigs have been established as a model for research of human diseases due to similarities, which includes size, organ structure and function, physiology and organ development [29, 30]. A high sampling rate (5 MS/s) for the differential probe was chosen to assure faithful reproduction of the signal in the nerve model. Extracellular recording of CAPs from peripheral nerve trunks is a well-established method to study nerve activity that is utilised both in teaching at universities and in preclinical studies [22–25]. A similar test method is used clinically e. g. by recording CAPs of the auditory nerve in cochlear implant patients (for a review see [31]). Another potential ex vivo test method suitable for this study could be a nerve-skeletal muscle preparation that encompasses the sciatic nerve and the associated gastrocnemius muscle [32–34]. This kind of preparation includes the neuromuscular junction and the effector or muscle allowing for a more direct measure of the output variable, i. e. muscle contraction, by means of electromyography or force measurement. However, this increases the overall complexity of the test method and the effort of dissection compared to the usage of an isolated peripheral nerve trunk. It would be much more challenging to standardise measurements at this level of complexity and there is no well-established ex vivo methodology incorporating a mammalian nerve-skeletal muscle preparation. Moreover, porcine nerves are very similar to human nerves regarding anatomy and physiology [29, 30]. Employing fresh tissue samples spares use of live animals, does not require animal testing application for the study, nor ethical animal husbandry or other additional efforts. Taken together, the porcine nerve trunk model seems to be the most parsimonious yet reliable ex vivo test method to address the aims of this study. A high inter-data set correlation (Fig. 3) demonstrates reproducibility of the outcome measure, which is due to the standardisation of the test method.

The justification for the two-stage setup is as follows: First, CAP recording during RF activation is not possible due to RF interference. Second, voltage recording enables multiple tests with the same stimulus in the nerve model, thereby demonstrating reproducibility. Regarding the position of the voltage measurement in the TURB model, we have pre-tested several positions. Only at a distance of ~ 3 mm from the RF application was the signal strength sufficient, and it was also far enough away not to damage the measuring electrode and theoretically a nerve [19]. The choice of position thus represents a clinical worst-case for nerve stimulation without nerve damage. In addition, the voltage recordings were processed (100 kHz low pass & scaling) to serve as input for the nerve model while minimising damage to the nerve samples and maximising nerve response. Therefore, the original strength of the voltage recordings was not the decisive parameter for the obtained NMS potentials. Instead, the scaling of the stimulation signals to adapt to the individual responsiveness and impedance of each nerve sample was crucial to obtain comparable NMS potentials across the entire measurement series. In parallel, scaling compensated for the reduced nerve excitability that occurs ex vivo during prolonged and repeated stimulations.

Another point of discussion is why generator voltage should not be used as input for a nerve model or for the immediate evaluation of NMS. At first glance, the voltage recordings of the TURB model look like an attenuated version of the generator voltage (data not shown). However, there are important differences: First, different tissue layers of the bladder attenuate the signal depending on frequency and thus act as a complex filter. Second, NMS may be caused by DC loops around the high-density or “active” electrode, which are caused by sparks and cannot be detected in the outer chain of generator, electrodes and connecting wires [35]. Thus, an application model with the relevant tissue components is necessary for the evaluation of NMS.

Regarding the outcome measure, we observed obvious differences between the RF generators in terms of their NMS potential (Fig. 4). Thus, the percentage of significant comparisons between the generators (~ 87%, Table 1) is an indicator for the selectivity of our test method. The differences in NMS potential have at least two reasons: First, differences of the effect settings, i. e. the minimum, default and maximum effect settings of each vendor are not in complete correspondence with respect to basic electrical parameters such as applied voltage and maximum power output. Second, differences in the programming of the RF generators and the components used in these generators could be the cause. Our test method is suitable for quantifying the NMS potential of different electrosurgical systems ex vivo but it does not reveal causes behind the differences observed. An additional study limitation is that the NMS potential does not translate into a relevant clinical parameter such as the incidence of obturator reflex or bladder perforation. However, the observed differences in NMS potential should clinically lead to more or less adverse NMS events. Of course, other influencing factors that are not represented by our model, such as the experience of the surgeon, patient-specific differences such as weight, pathological changes, etc. must also be taken into account. Furthermore, clinically investigated influencing factors, such as the monopolar versus bipolar technique or the use of a pharmacological block, were not addressed in our ex vivo model. These system-level questions are likely to remain a task of in vivo testing. Nonetheless, the comparison of new electrosurgical systems with well-established and safe equipment allows for a meaningful safety evaluation. To our knowledge, the test method described here is the first to address the ex vivo risk assessment of NMS for transurethral resection devices. In the future, this test method can easily be adapted to test bipolar TURP as well as monopolar TURB and TURP, e.g. the TURB model can be adapted to emulate TURP using prostatic instead of bladder tissue.

## Conclusions

Our test method is suitable for quantifying the NMS potential of different electrosurgical systems ex vivo with high selectivity at a reasonable degree of standardisation and with justifiable effort. Our results suggest that the clinical incidence of NMS is considerably influenced by the type of RF generator. The development of future generations of RF generators takes advantage from the proposed test standard through higher safety and by replacement or reduction of the extent of animal testing. Most important, health professionals and treated patients will benefit from improved RF surgery using generators with a low risk of NMS.

## Supplementary information

**Additional file 1: Figure S1.** Automated CAP detection required that a predefined voltage threshold of 300 μV was exceeded - defining the onset of the CAP, a minimum peak CAP amplitude of 350 μV was reached within 1 ms after the onset, a zero transition - marking the onset of the second phase of the CAP - was detected within 1.3 ms after the onset and that the second phase of the CAP lasted at least 300 μs, i. e. continuously negative voltage. The area under the first phase of each CAP (blue area) was calculated by spline interpolation (Scilab function ‘intsplin’) from the detected onset to the zero transition. This area represents the individual CAP strength.

## Data Availability

The data that support the findings of this study are available from Erbe Elektromedizin GmbH but restrictions apply to the availability of these data, which were used under license for the current study, and so are not publicly available. Data are however available from the authors upon reasonable request and with permission of Erbe Elektromedizin GmbH.

## Abbreviations

CAP: (Evoked nerve) Compound action potential
NMS: Neuromuscular stimulation
RF: Radio-frequency
TURB: Transurethral resection of bladder (tumour)
TURP: Transurethral resection of the prostate
