# Supplementary figures and images for: Risk assessment of neuromuscular stimulation by energy-based transurethral resection devices: an ex vivo test standard
Source: BMC Urol. 2020 May 27;20:59. doi: 10.1186/s12894-020-00630-5 (PMC7254752; doi:10.1186/s12894-020-00630-5)

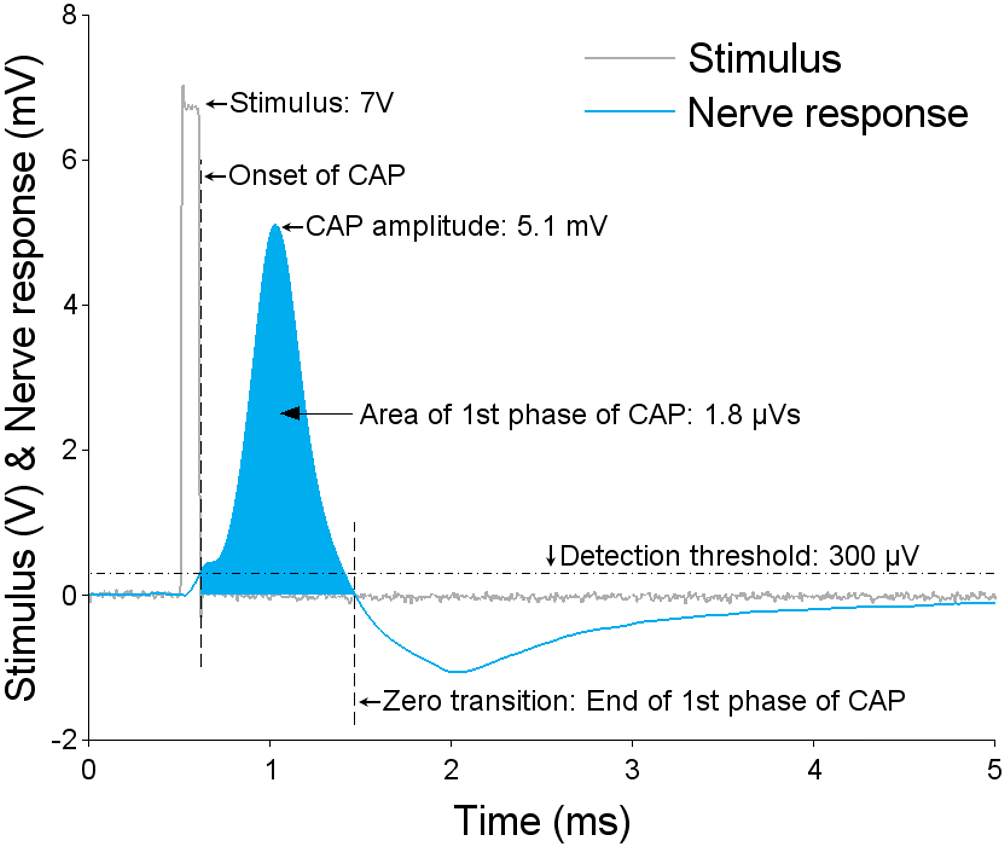

Supplement: Supplementary file 1 — Additional file 1: Figure S1. Automated CAP detection required that a predefined voltage threshold of 300 μV was exceeded - defining the onset of the CAP, a minimum peak CAP amplitude of 350 μV was reached within 1 ms after the onset, a zero transition - marking the onset of the second phase of the CAP - was detected within 1.3 ms after the onset and that the second phase of the CAP lasted at least 300 μs, i. e. continuously negative voltage. The area under the first phase of each CAP (blue area) was calculated by spline interpolation (Scilab function ‘intsplin’) from the detected onset to the zero transition. This area represents the individual CAP strength. [file 12894_2020_630_MOESM1_ESM.tif]
